# A Curious Gingival Erosion: A Rare Oral Manifestation of Fixed Drug Eruption

**DOI:** 10.7759/cureus.108954

**Published:** 2026-05-16

**Authors:** Claire M Lutz, François Le Pelletier de Glatigny, Anne-Laure Ejeil

**Affiliations:** 1 Department of Odontology, Oral Medicine and Oral Surgery, Bretonneau Hospital, Assistance Publique-Hôpitaux de Paris (AP-HP) Université Paris Cité, Paris, FRA; 2 Department of Pathology, Pitié-Salpêtrière Hospital, Assistance Publique-Hôpitaux de Paris (AP-HP) Université Paris Cité, Paris, FRA

**Keywords:** adverse event, drugs, oral diseases, oral mucosa, toxidermias

## Abstract

Fixed drug eruption (FDE) is a drug-induced, immune-mediated cutaneous reaction characterized by recurrent, well-demarcated, erythematous, and edematous lesions that reappear at the same site upon re-exposure to the offending drug. Mucosal involvement in FDE is relatively rare and clinically distinct. Oral FDE often presents differently from its cutaneous counterpart, which can complicate diagnosis.

We report the case of a 35-year-old man, allergic to penicillin and metronidazole, who presented with an isolated erosion on the vestibular attached gingiva of the upper right molar region. Clinical examination revealed no lymphadenopathy or other lesions in the oral cavity, and histopathological analysis showed lichenoid inflammation. After initial clinical evaluations, the patient later revealed he had experienced similar lesions following the intake of paracetamol, including episodes affecting the head of the penis. The lesion resolved spontaneously within 15 to 21 days after discontinuation of paracetamol.

Based on the patient's history, clinical presentation, and histopathological findings, a diagnosis of FDE was made. This case highlights the importance of a thorough drug history in diagnosing oral FDE, as well as the atypical clinical presentation of FDE in the oral cavity compared to its cutaneous form. Although rare, oral FDE should be considered in cases of recurrent, well-demarcated erosions associated with specific drug exposures. Early recognition and avoidance of the causative drug are essential for effective management.

## Introduction

Fixed drug eruptions (FDE) are a relatively common type of immunoallergic toxidermias triggered by the administration of a drug. These lesions are characterized by reappearance at the same site upon re-exposure and are often observed on the skin. These clinical manifestations typically affect the trunk or limbs. They are often asymptomatic and spontaneously resolve, leaving post-inflammatory hyperpigmentation in about 30% of cases. While an increasing number of drugs are being implicated, the main families responsible for FDE remain analgesics and antibiotics [1]. While cutaneous FDE is common, mucosal involvement, particularly in the oral cavity, is rarer and can present diagnostic challenges. Clinically, oral FDE manifests as asymptomatic erosions or ulcerations, often mistaken for other oral conditions such as lichen planus or traumatic ulcers. Early recognition and management of these lesions are crucial to prevent recurrences, misdiagnoses, and potential aggravation [2].

In clinical practice, the diagnosis of FDE relies mainly on patient history, recurrence at the same anatomical site following drug exposure, and clinicopathologic correlation, while drug provocation testing is rarely performed due to safety concerns.

This report describes a case of an isolated gingival erosion, identified as FDE following the administration of paracetamol, highlighting the importance of accurate diagnosis and the need for a thorough drug history in cases of unexplained oral ulcerations.

## Case presentation

**Figure 1:**
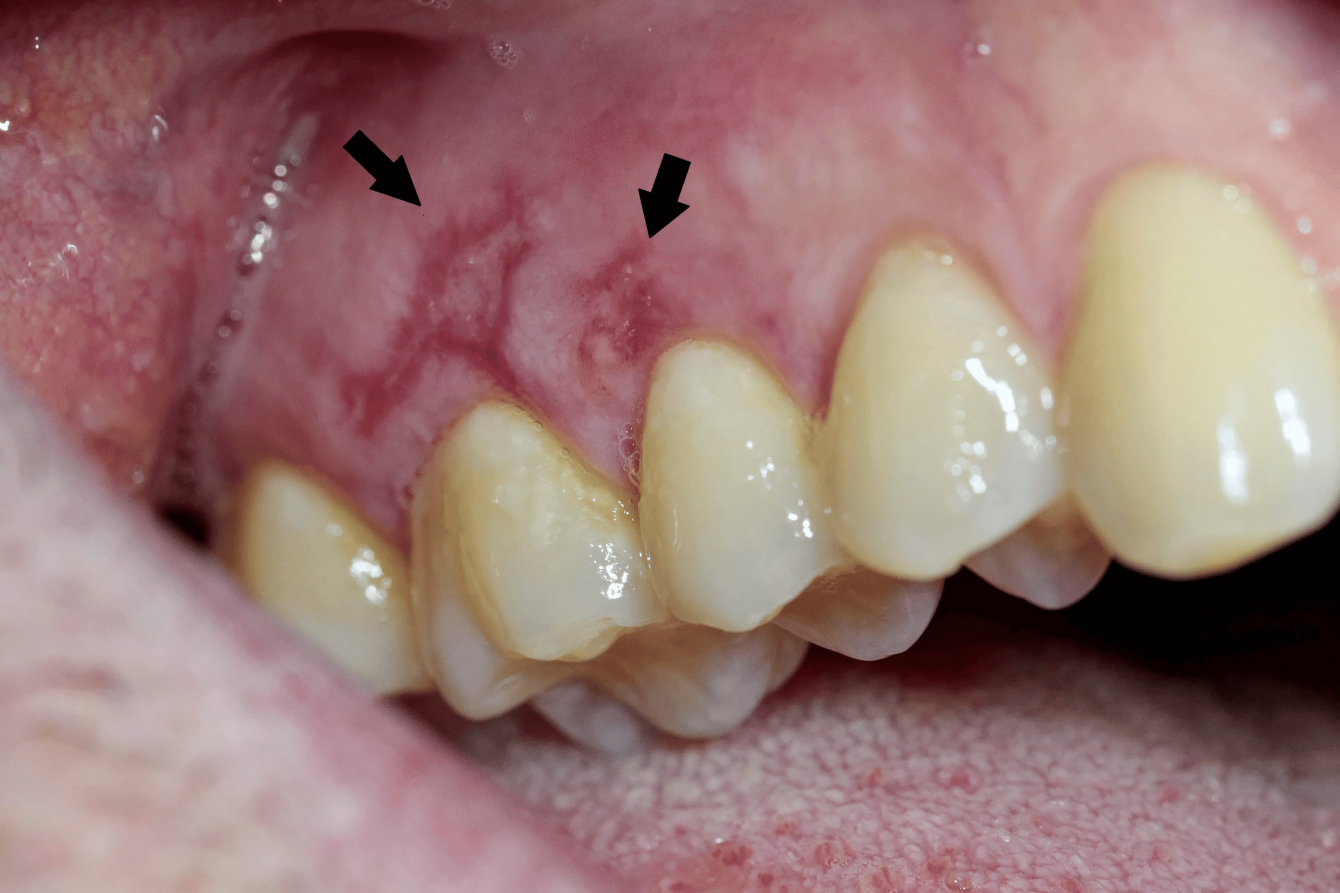
Clinical appearance of fixed drug eruption: well-delimited erosion located on the attached gingiva of the right maxillary second premolar and first molar (arrows)

A 35-year-old man, with a history of penicillin and metronidazole allergy, was referred for an oral pathology consultation at Bretonneau Hospital by the oral emergency department for an upper right "gingival sore", associated with mild pain. The lesion had initially appeared a few days prior as a centimetric, well-demarcated erosion on the vestibular attached gingiva of the upper right first molar. Routine blood tests and screening for sexually transmitted infections were performed, given the history of a similar lesion involving the glans penis. All results were unremarkable, helping to exclude infectious causes. By the time of his follow-up in the oral pathology department, no spontaneous healing had occurred, and the lesion had extended to involve the gingiva of the upper right second premolar as well (Figure 1).

The patient's history and clinical examination findings excluded trauma or burn as possible etiologies. Clinical examination revealed no associated lymphadenopathy, and no other lesions were noted.

A biopsy was performed, revealing a lichenoid inflammatory pattern. Histopathologic examination showed a focal elongation of a single epithelial rete ridge, which was insufficient to support a diagnosis of verrucous carcinoma. The inflammatory infiltrate was predominantly lymphocytic but not arranged in a band-like pattern, and its lower border was ill-defined, arguing against oral lichen planus. The epithelial-connective tissue junction appeared focally blurred. After the biopsy, a topical cream containing xylocaine was prescribed to alleviate post-procedural pain.

**Figure 2:**
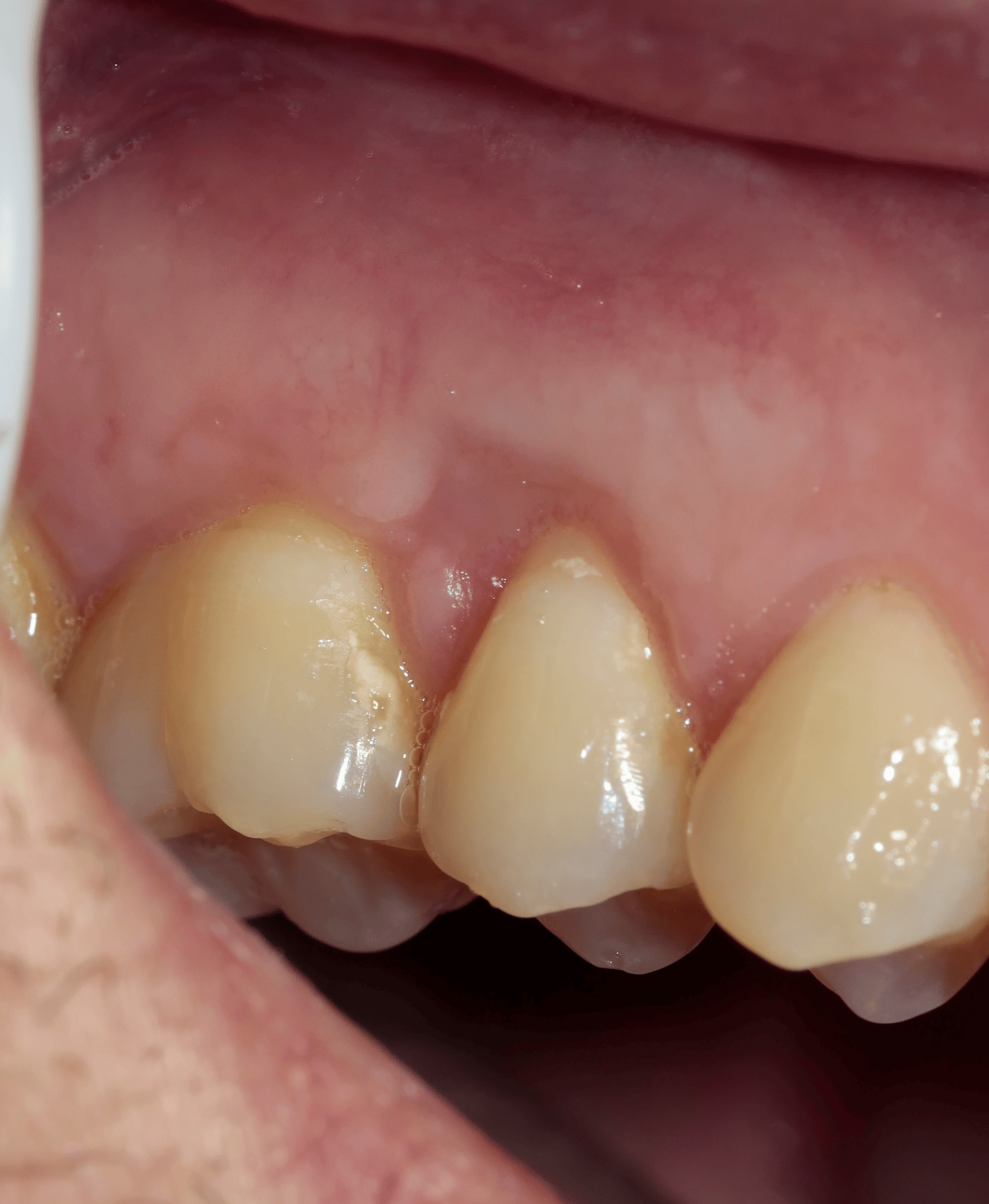
Clinical characteristics of fixed drug eruption after healing of the biopsy site, showing complete healing of the attached gingiva in 15 days

Two weeks later, at the post-biopsy follow-up visit, the erosion had fully healed, prompting a reevaluation of the histological diagnosis of lichen planus, as this condition was clinically inconsistent and lacked other typical locations of involvement (Figure 2).

During this consultation, the patient disclosed that he had experienced similar episodes in the past, including involvement of the glans penis, following the intake of paracetamol.

Based on the patient’s medical history, the recurrent nature of the lesion, and a joint discussion with the pathologist, the diagnosis of an isolated oral manifestation of FDE likely induced by paracetamol was made. No additional treatments were administered since the lesion had healed in 15 days. The patient was advised to avoid paracetamol to prevent future recurrences. Although follow-up was recommended in the event of symptom recurrence, we did not receive further reports. The absence of recurrence may reflect successful avoidance of paracetamol. However, loss of follow-up cannot be excluded.

## Discussion

This case underscores the diagnostic challenges posed by oral manifestations of FDE, a condition more frequently observed with cutaneous involvement. The diagnosis of FDE in this instance was reached following a comprehensive evaluation encompassing the patient's medical history, the clinical presentation of the lesion, and histopathological findings.

FDE is classified as a localized immunoallergic drug toxidermia. While its exact pathogenesis remains incompletely understood, accumulating evidence highlights the pivotal role of resident memory CD8+ T cells (TRM). Following initial sensitization to a drug, subsequent re-exposure rapidly activates TRM cells at the same site, leading to a localized inflammatory response [3]. Although CD8+ T cells are the primary mediators of this process, regulatory T cells are believed to modulate the immune response, contributing to the self-limiting nature of FDE by controlling excessive inflammation [3,4]. This immune reaction can be triggered by an increasing number of molecules such as tamsulosin, piroxicam, or tinidazole [5-7]. However, analgesics and antibiotics remain the primary implicated drug families, with paracetamol being one of the most commonly involved agents [2,8]. The reported frequency of FDE varies widely across studies, ranging from approximately 10% to nearly 50% of cutaneous adverse drug reactions [1].

FDE is generally characterized by the eruption of erythematous macules or bullae a few days after exposure to the causative agent. These lesions tend to recur at the same site upon re-exposure, most frequently on the trunk or limbs [1,9]. Oral involvement, while less common than cutaneous lesions, has been reported, typically presenting as erosions, aphthoid ulcerations, lichenoid-like lesions, or erythematous patches [2,3].

In the present case, the recurrence of a geometric erosion on the gingiva following paracetamol intake, coupled with the patient's report of a similar event involving the glans penis, strongly suggested FDE. No other concomitant medications or potential triggers were identified during the episodes. Lesions on the glans penis are frequently observed in men with FDE, either in isolation or in association with oral mucosal involvement [4,10]. As observed in this case, oral FDE manifestations are often asymptomatic and resolve spontaneously within a few days to weeks. However, the absence of pigmentation sequelae in the oral cavity contrasts with the more typical pigmentation seen in cutaneous FDE, which occurs in up to 30% of cases [2,8].

This case highlights an atypical presentation of FDE, with lesions localized to the attached gingiva, a less commonly affected site in oral FDE. As noted by Mortazavi (2022), the most frequent sites of oral FDE include the lips, tongue, and buccal mucosa, with gingival involvement being relatively rare [8]. This unusual presentation further emphasizes the diagnostic complexity of oral FDE, which may lead to diagnostic delays or misdiagnosis, particularly in the absence of accompanying cutaneous lesions [6].

Diagnosing FDE, particularly in the oral cavity, can be challenging. The lesions often present with nonspecific features that mimic other conditions such as lichen planus, autoimmune bullous diseases, or even Behçet's disease [2]. Infectious causes, such as herpes simplex virus or sexually transmitted infections, were also evaluated but excluded based on clinical presentation and negative investigations. Autoimmune and inflammatory conditions, including oral lichen planus and bullous diseases, were also explored; however, the absence of characteristic histopathologic features, spontaneous healing, and the recurrent, site-specific nature of the lesions argued against these diagnoses. Traumatic ulcers were also considered but were unlikely based on the patient’s history, as no local traumatic factor was identified. In this case, the histopathological findings were crucial in ruling out other potential diagnoses suggested by the lesion’s persistent and initially progressive nature. The histological similarity between FDE and lichen planus, both of which can present with lichenoid-type inflammation, underscores the importance of correlating clinical history with biopsy results to reach an accurate diagnosis [2,3].

Furthermore, Brahmi (2016) highlights that FDE cases often present in older adults, with women being more frequently affected in some cases of cutaneous FDE [11]. This aligns with the demographic data observed across broader FDE studies and may serve as a reminder of the importance of comprehensive patient evaluation, especially in atypical presentations.

Although drug provocation testing can be useful in confirming drug-induced eruptions, it was not performed in this case due to ethical and safety considerations, as re-exposure could have induced a more severe reaction. The diagnosis was therefore based on clinical history, including a clear temporal relationship, recurrence at the same site, and complete resolution after drug withdrawal.

The primary management of FDE involves the avoidance of the causative drug [8]. In some cases, antihistamines or topical corticosteroids may be used to alleviate symptoms, though the condition is largely self-limiting [12]. Consistent with the self-limiting nature of FDE, the lesion in this case resolved spontaneously within 15 to 21 days without further intervention once paracetamol was discontinued. Screening for cross-reactivity with different drugs from the same class can be considered to prevent future episodes [3].

The differential diagnosis of oral erosion is broad and includes numerous conditions that present similar clinical features. However, this case emphasizes the importance of a thorough drug history and clinical follow-up to establish the FDE diagnosis. The patient’s report of similar lesions on the glans penis following paracetamol intake further supports the diagnosis and highlights the potential for FDE to involve multiple mucosal sites [11]. Mortazavi's comprehensive data demonstrates that mucosal FDE lesions can manifest in isolation or in association with other sites, supporting the multi-site involvement observed in this case [11]. As noted, multi-mucosal involvement may serve as an important diagnostic clue, particularly in recurring cases where other conditions have been excluded [10]. While FDE typically presents as well-demarcated erythematous macules or erosions, its clinical spectrum can vary. Additionally, a more severe form of FDE, characterized by the appearance of hemorrhagic bullae over extensive areas, including the oral cavity, can be mistaken for other severe drug-induced reactions such as Stevens-Johnson syndrome (SJS) or toxic epidermal necrolysis (TEN). The distinction between these entities is critical, as FDE generally lacks the systemic involvement observed in SJS/TEN and follows a more benign course [3].

## Conclusions

This case underscores the diagnostic complexity of oral FDE, a condition often overlooked due to its highly variable and nonspecific presentations, ranging from mild erythematous patches to painful bullous or erosive lesions that may mimic autoimmune blistering disorders, infectious stomatitis, oral lichen planus, or even neoplastic processes. While rare, oral FDE should be systematically considered in patients with recurrent mucosal lesions, particularly when lesions reappear at the same site following re-exposure to suspected medication. In this patient, the temporal association between drug intake and lesion recurrence, along with the fixed, well-demarcated nature of the erosions, strongly supported the diagnosis. Prompt discontinuation of the offending agent led to complete resolution within 15 days, which is consistent with the self-limiting nature of the condition and supports the importance of a detailed drug history in patients with unexplained oral lesions. Notably, the absence of cutaneous involvement further highlights the potential for misdiagnosis, as oral FDE may be mistaken for more severe or chronic conditions.
